# Arthroscopic Debridement and Proximal Fibular Osteotomy in the management of knee Osteoarthritis: A Descriptive Longtudinal Study

**DOI:** 10.31729/jnma.8778

**Published:** 2024-10-31

**Authors:** Vijayendra Adhikari, Prasamsha Sitaula, Ojas Thapa, Sumi Singh, Praphulla Shrestha, Pralhad Kumar Chalise, Anil Kumar Mishra, Ramesh Prasad Singh

**Affiliations:** 1 Department of Orthopaedics, Nepal Medical College and Teaching Hospital, Kathmandu, Nepal; 2 Nepalese Army Institute of Health Sciences College of Medicine, Kathmandu, Nepal; 3 School of Medicine and Biomedical Sciences, University of New York, Buffalo, USA

**Keywords:** *knee osteoarthritis*, *arthroscopic debridement*, *proximal fibular osteotomy*

## Abstract

**Introduction:**

Knee osteoarthritis commonly occurs in the medial compartment. Arthroscopic debridement eases pain by irrigating debris and inflammatory cytokines. Proximal fibular osteotomy shifts the loading force more laterally from the medial compartment, eliminating pain and delaying degenerative progression. This study aims to evaluate pain relief by combining both procedures in knee osteoarthritis of the medial compartment.

**Methods:**

The study was conducted in Nepal Medical College Teaching Hospital from November 2021 to December 2022 after the ethical approval from institutional review committee (IRC Ref. No. 025-078/079). The outcome was assessed by visual analog score and knee society scores, who underwent a combined procedure of arthroscopic debridement and proximal fibular osteotomy during a study period of 12 months.

**Results:**

Diagnostic arthroscopy was done in 32 patients, out of which, 27 patients underwent combined procedure with arthroscopic debridement and proximal fibular osteotomy. The mean visual analog score in combined procedure was 6.89±0.93 initially and 3.11±0.69 at 12 months follow up. The mean knee society score in combined procedure was 46.85±6.1 preoperatively and 84.26±8.27 postoperatively at 12 months follow up. Common peroneal nerve neuropraxia was observed in 8 (29.62%) patients, which resolved spontaneously during 6 months of time period.

**Conclusions:**

Combined arthroscopic debridement and proximal fibular osteotomy procedure is effective in treating knee osteoarthritis of the medial compartment, as it decreases pain and improves knee society score.

## INTRODUCTION

Osteoarthritis of knee is chronic, progressive disease causing joint pain, stiffness and deformity with radiological findings of subchondral bone sclerosis, decreased joint space and cyst formation in weight bearing areas.^1,2^ Radiographic and symptomatic prevalence rate is estimated to be 37% and 12% respectively in elderly persons above 60 years age.^3^

It commonly occurs in the medial compartment, bearing 60-80% of the load.^4^ The load shifts medially due to non-uniform settlement and degeneration, resulting in varus knee and aggravating osteoarthritis progression.^5^ Surgical treatments include options like high tibial osteotomy, proximal fibular osteotomy, unicompartmental knee arthroplasty and total knee arthroplasty.^6^ Arthroplasties are complex and expensive, not ideal for young patients with mild to moderate osteoarthritis.^6,7^ Proximal fibular osteotomy shifts the load laterally, relieving pain and delaying degeneration.^5^ Arthroscopic debridement eases pain by removing inflammatory cytokines and debris.^8^

Therefore, the study aims to evaluate if combining procedure arthroscopic debridement with proximal fibular osteotomy offers pain relief and improves joint function with fewer complications.

## METHODS

This prospective study was conducted in the department of Orthopaedics of Nepal Medical College and Teaching Hospital, Nepal, from November 2021 to December 2022. The study population included all patients who underwent arthroscopic debridement and proximal fibular osteotomy during the study period. The inclusion criteria for the surgery were knee osteoarthritis of the medial compartment and having clinical symptoms like pain and deformity of the knee. The radiological criteria for inclusion were the presence of a collapsed medial joint space and a maintained lateral joint space on standard weight bearing x-ray of the knee, anterior posterior and lateral views. Ethical approval for the study was obtained from the institutional review committee of Nepal Medical College (IRC ref. No. 025-078/079).

The osteoarthritis of knee was graded according to Kellgren-Lawrence criteria radiologically,^9^

Grade 1 - doubtful joint space narrowing and possible osteophytic lipping.

Grade 2 - moderate multiple osteophytes, definite narrowing of joint space, some sclerosis and possible deformity of bone ends.

Grade 3 - moderate multiple osteophytes, definite narrowing of joint space, some sclerosis and possible deformity of bone ends.

Grade 4 - large osteophytes, marked narrowing of joint space, severe sclerosis and definite deformity of bone ends.

The medial and lateral joint space of the knee on x-ray were measured manually and then patients were evaluated with visual analog score and the Knee Society score^10^ pre and post operatively to evaluate the functional outcome clinically and radiologically. Knee society score evaluates the clinical and functional score. The clinical score is based on pain, range of motion, alignment, stability and the functional score is based on the patient's ability to perform daily activities like walking, climbing stairs and performing overall function. The higher knee society score indicates better outcomes and lower levels of pain and dysfunction.

After spinal anesthesia, all the patients were kept in supine position and tourniquet inflated. The surface marking of patella, tibial tubercle, patellar tendon, fibula, the medial and lateral tibial joint line were marked. Diagnostic arthroscopy was performed through conventional arthroscopic portals, to assess integrity of the articular cartilage of patella-femoral, lateral compartment and medial compartment of the knee. Where it was confirmed that the medial femoral condyle cartilage was eroded or broken down and that the lateral femoral condyle cartilage was good enough to proceed with Proximal Fibular Osteotomy. After diagnostic arthroscopy, all patients underwent joint debridement and lavage. Those with intact lateral femoral condyle cartilage knees underwent Proximal Fibular Osteotomy. Through the posterolateral approach of the fibula, subcutaneous tissue was dissected, the intermuscular space between the peroneal and soleus muscles was separated to reach the proximal fibula and subperiosteal dissection was performed. The osteotomy site for fibula of about 2 cm long was marked 6-10 cm away from the fibular head according to the height of the patient with a micro oscillating saw, 2 cm bone block was cut and removed. Hemostasis was maintained, closure was done and dressing was applied.

The patients were advised for quadriceps strengthening exercises and full weight bear as pain tolerable from the next day of surgery. Postoperative weight-bearing X-rays of the knee were done, where the lateral and medial joint space were measured manually and visual analog score with knee Society score was evaluated and recorded at the 3 month, 6 month and 12 months.

The collected data were analyzed using software SPSS.

## RESULTS

There were 32 patients with knee osteoarthritis of the medial compartment. All patients underwent diagnostic arthroscopy among which, 5 patients had a cartilage erosion in the lateral femoral condyle, who then underwent debridement only, and hence, were excluded from the study. The combined procedure with arthroscopic debridement and proximal fibular osteotomy was done in the remaining 27 patients. Out of those 27 patients, 19 (70.38%) were male and 8 (29.62%) were female. Their mean age was 46.97±4.39 years. Right knee involvement was in 15 (55.55%) patients and left knee was involved in 12 (44.45%) patients. Osteoarthritis was classified according to Kellgren - Lawrence criteria ^9^, 19 (70.37%) had grade 2 while 7 (25.92%) had grade 3 and 1 (3.70%) had grade 4 knee osteoarthritis.

The mean visual analog score in combined procedure (n=27) decreased from 6.89±0.93 initially to 3.11±0.69 at the final follow-up. Postoperative weight-bearing X-rays measured manually showed visual improvement in the medial joint space of the knee in 25 cases of Grade 2 and 3, while no change was observed in 2 cases of grade 3 and grade 4.

There was significant improvement of mean knee society score^10^ in combined procedure from 46.85±6.1 preoperatively to 84.26±8.27 postoperatively at 12 months follow up (Table 1).

**Table 1:** Clinical outcome of patient undergoing arthroscopic debridement and proximal fibular osteotomy in the management of knee Osteoarthritis (n=27)

	Pre operatively	3 Month	6 Month	12 Month
visual analog score	6.89 ± 0.93	5.52 ± 0.93	4.04 ± 0.94	3.11 ± 0.69
knee society score	46.85 ± 6.1	63.93 ± 6.8	74.41 ± 8.20	84.26 ± 8.27

Eight patients (29.62%) experienced neuropraxia of the common peroneal nerve, which resolved spontaneously within 6 months. Post-operative complications like wound infection or neurovascular injury were not observed.

## DISCUSSION

Knee Osteoarthritis is a degenerative joint disease affecting the elderly population, where inflammatory components and mechanical abnormalities leads to pain and deformity of the knee joint due to breakdown of articular cartilage and subchondral bone leading to difficulty in daily activities.^11^ Initially knee osteoarthritis is involved in medial compartment, followed by the lateral and patellofemoral compartments of joint.^4^ The primarily aim for the treatment of knee osteoarthritis of medial osteoarthritis is to delay the course of the disease and relieve symptoms which includes oral medications, intra-articular injections and surgical interventions like proximal fibular osteotomy, high tibial osteotomy, unicompartmental or total knee arthroplasty, along with physiotherapy and lifestyle modifications.^5^ All treatment modalities have their pros and cons, which have a significant impact on daily activities.

Yang ZY et al^5^ reported after proximal fibular osteotomy, pain is decreased, correction of varus deformity and slowing down the progression of degenerative changes in joint is due to shifting of loading force from medial to more laterally after the osteotomy of the proximal fibula. Zuber et al^12^ have concluded that combined arthroscopic debridement and proximal fibular osteotomy in Kellgren and Lawrence Grade I-III knee osteoarthritis, effectively decrease pain and correct varus deformity. The comparative study of arthroscopic debridement versus proximal fibular osteotomy combined with arthroscopic debridement by Hao L et al.^13^ found that combined techniques result in both immediate and long-term pain relief, improved knee society score, slowdowns the cartilage degeneration and improves varus deformity. Abdelsamie M et al.^14^ stated that a combined procedure with proximal fibular osteotomy and arthroscopic joint debridement decreases varus deformity and improves symptoms.

In the present study, the visual analog score preoperatively was 6.89±0.93 which decreased to 3.11±0.69 after 12 months. Similarly, in Zuber M et al. 12 study, the visual analog score decreased significantly from 6.66±1.24 preoperative to 2.33±0.98 postoperatively and the Janjua FA et al.^15^ study shows visual analog score after 1 week to be 7.47+0.991 which then decreased to 5.23±1.25 after 3 months in combined procedure with arthroscopic debridement and proximal fibular osteotomy.

The knee Society Score in our study was 46.85±6.1 preoperatively which increased to 84.26±8.27 at 12 months. Similarly, in Zuber M et al.^12^ study, knee society score increased from 55.6±11.54 preoperatively to 74.66±12.54 postoperatively and Janjua FA et al15 study shows that the knee society score after 1 week was 46.32±12.28 which then increased to 58.22±13.92 after 3 months. Tian J et al^16^ performed systematic review and meta-analysis to evaluate the therapeutic effects of arthroscopic debridement and proximal fibular osteotomy in medial knee osteoarthritis. Ten studies analyzed visual analog score in 687 patients, among them 350 cases were operated with combined procedure of arthroscopic debridement and proximal fibular osteotomy and the remaining 337 cases were control group. They observed that the visual analog score was lower in the combined procedure group. Similarly, six studies analyzed knee society scores in 455 patients with 236 cases were operated with combined procedure of arthroscopic debridement and proximal fibular osteotomy and 219 were control. Knee society score of the combined procedure group were higher than the control group.

Wang X et al^17^ observed postoperatively improvement of medial knee joint space compared to preoperatively in weight bearing x-ray, whereas in our study too there was slight improvement of medial knee joint space postoperatively, especially in grade 2 knee osteoarthritis.

In review study of Vaish A et al^18^ on proximal fibular osteotomy for knee osteoarthritis, they found 14 cases of transient nerve palsy out of 294 cases, among them, superior peroneal nerve palsy in 12 patients and common peroneal nerve palsy in 2 patients were seen. All nerve palsies recovered within average time of 11.6 months. Similarly, in our study eight patients experienced neuropraxia of the common peroneal nerve, which resolved spontaneously within 6 months.

Yang ZY et al^5^ reported four cases of proximal fibular osteotomy converted to total knee arthroplasty after 12 months of follow up, whereas in our study after combined arthroscopic debridement and proximal fibular osteotomy none of the patients underwent second surgery. Similarly, Lu et al^19^ also reported no conversion to total knee replacement following proximal fibular osteotomy.^21^

In our study, after the combination technique of arthroscopic debridement with proximal fibular osteotomy, the visual analog score and knee Society Score improved significantly between initially and final follow-up without any significant complications.

High tibial osteotomy is recommended in young patients having a knee osteoarthritis of medial compartment, it corrects the knee alignment and delay the course of the disease but major drawbacks are full weight bearing is prolonged, wound infection, paralysis of peroneal nerve and high chances of delayed union or nonunion.^4,20-22^

In later stage of knee osteoarthritis, especially in older patients, arthroplasty effectively eliminate pain and knee joint function gets better but main disadvantages are it is expensive, complex surgery and some of them may need a second revision surgery.^6,23^

Surgical interventions like High tibial osteotomy or arthroplasty, both relieves pain and correct deformity but they have their own limitations, such as being technically demanding procedures, the risk of iatrogenic fractures and the need for additional implant insertion. The high cost of implants and surgery is a major concern, so alternative procedures that are simple, cost-effective, provide good functional outcomes and improve daily activities are needed. The literature has reported that arthroplasty after failed high tibial osteotomy has surgical challenges and less satisfactory outcomes compared to without prior osteotomy procedure like proximal fibular osteotomy.^24^

The simple minimal invasive surgery, proximal fibular osteotomy needs no additional implant insertion which reduces pain, increase joint space and delays degenerative progression by shifting the loading force due to mechanism of non-uniform settlement.^25^

The crucial factor that leads to non-uniform settlement is due to support of the fibula to the lateral condyle, mechanical axis is shifted medial due to absence of support to medial condyle causing varus deformity and progression of the medial compartment of the knee joint is aggravated.^5^

Arthroscopic debridement with Proximal fibular osteotomy in medial joint osteoarthritis of the knee involves the dilution of inflammatory mediators and the removal of loose bodies and cartilage debris through arthroscopic debridement^8^ and Proximal fibular osteotomy eliminates pain, correct deformity and increased joint space as osteotomy of proximal fibula causes the weakening of lateral condyle support leading to shift of loading force from the medial compartment to more laterally.^5^

The limitations of this study were a smaller number of patients, additionally, the study follow-up period was relatively short, limiting the ability to evaluate the long term outcomes and durability of the combined arthroscopic debridement and proximal fibular osteotomy procedure in treating osteoarthritis. Lack of a control group is another limitation, as it prevents a direct comparison between the combined procedure and other treatment modalities, such as high tibial osteotomy or total knee arthroplasty. Moreover, the study relied on manual measurements of joint spaces and subjective scoring systems, which could introduce observer bias and affect the accuracy of the results. Finally, the single center design of the study may limit the applicability of the findings to different clinical settings and patient populations.

## CONCLUSIONS

The combined arthroscopic debridement with proximal fibular osteotomy procedure is effective in treating knee osteoarthritis of the medial compartment as it decreases pain and improves mean knee society score.
